# Global nickel anomaly links Siberian Traps eruptions and the latest Permian mass extinction

**DOI:** 10.1038/s41598-017-12759-9

**Published:** 2017-09-29

**Authors:** Michael R. Rampino, Sedelia Rodriguez, Eva Baransky, Yue Cai

**Affiliations:** 1 Department of Biology, New York University, New York, NY 10003 USA; 2 Department of Environmental Studies, New York University, New York, NY 10003 USA; 3 NASA, Goddard Institute for Space Studies, New York, NY 10025 USA; 4 Department of Environmental Science, Barnard College, New York, NY 10027 USA; 5 Lamont-Doherty Earth Observatory of Columbia University, Palisades, NY 10964 USA

## Abstract

Anomalous peaks of nickel abundance have been reported in Permian-Triassic boundary sections in China, Israel, Eastern Europe, Spitzbergen, and the Austrian Carnic Alps. New solution ICP-MS results of enhanced nickel from P-T boundary sections in Hungary, Japan, and Spiti, India suggest that the nickel anomalies at the end of the Permian were a worldwide phenomenon. We propose that the source of the nickel anomalies at the P-T boundary were Ni-rich volatiles released by the Siberian volcanism, and by coeval Ni-rich magma intrusions. The peaks in nickel abundance correlate with negative δ^13^C and δ^18^O anomalies, suggesting that explosive reactions between magma and coal during the Siberian flood-basalt eruptions released large amounts of CO_2_ and CH_4_ into the atmosphere, causing severe global warming and subsequent mass extinction. The nickel anomalies may provide a timeline in P-T boundary sections, and the timing of the peaks supports the Siberian Traps as a contributor to the latest Permian mass extinction.

## Introduction

The end-Permian mass extinction (252 Myr ago) was the most severe in the geologic record, devastating both marine and terrestrial fauna and flora^1^. The Global Stratigraphic Section and Point (GSSP) for the Permian/Triassic boundary is the Meishan section in southeast China^2^. The latest Permian at Meishan is marked by a negative shift in carbon-isotope values in carbonates and organic carbon, which is seen worldwide in P-T boundary sections^3^. The carbon-isotope anomaly has been attributed by some to the release of volcanogenic carbon dioxide and methane into the atmosphere and oceans, from the Siberian Traps eruptions, and from explosive interactions of magma intruding carbon-rich sediments^4,5^.

**Figure 1:**
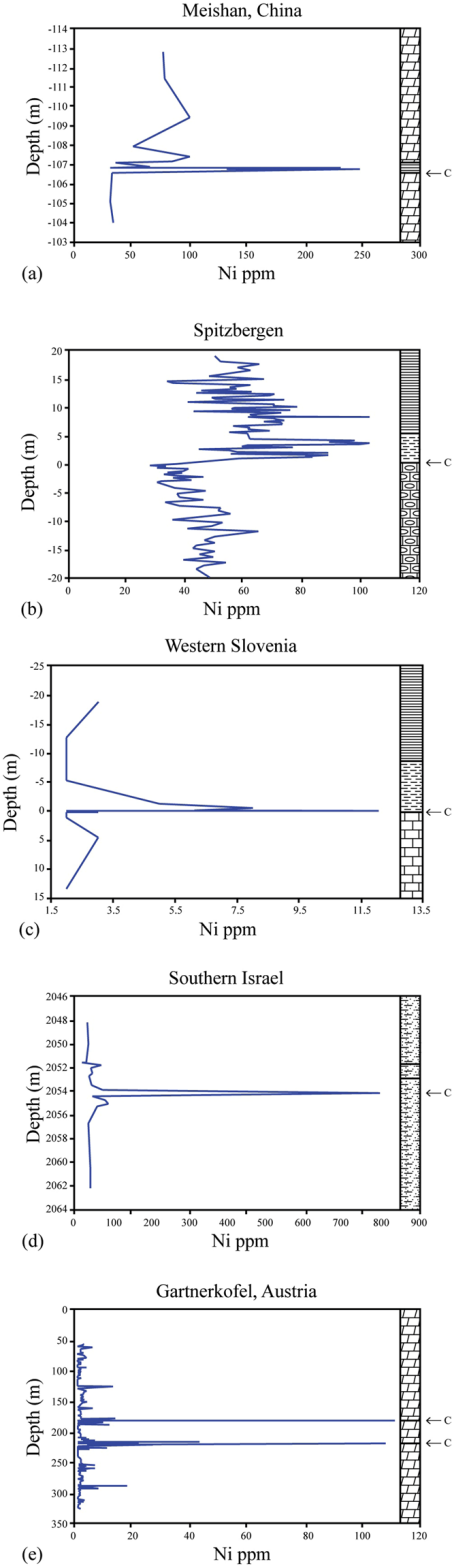
Nickel concentrations in latest Permian sediments. C marks the position of the latest Permian negative shifts in δ^13^C. (**a**) Meishan, China (zero is the latest Permian mass extinction level) (17 pts.); (**b**) Spitzbergen (zero is the end-Permian extinction level) (123 pts.); (**c**) Western Slovenia (zero is the latest Permian extinction level) (14 pts.); (**d**) Southern Israel (the nickel anomaly is just below the fungal event and the negative shift in δ^13^C that mark the P-T boundary)^9^ (18 pts.); (**e**) The Gartnerkoefel GK-1 core from the Carnic Alps in Austria (positions of nickel anomalies match two negative δ^13^C anomalies that bracket the P-T boundary)^12^ (305 pts.).

Kaiho *et al*.^6^ reported a spike of nickel abundance (up to about 90 ppm, over a background of about 20 ppm) at the Permian-Triassic GSSP, coincident with the latest Permian negative carbon-isotope shift, and extinction level. A more recent study by Rothman *et al*.^7^ found nickel concentrations in the latest Permian at Meishan up to 250 ppm over a background of around 40 ppm (on a carbonate-free basis) at the same stratigraphic level (Fig. 1a). The Shangsi section in southern China shows a similar Ni anomaly (92 ppm over a background of less than ~30 ppm) near the latest Permian extinction level^8^. In addition to the Chinese sections, a search of the literature turned up similar nickel-abundance anomalies at the end of the Permian in Israel^9^; Western Slovenia^10^; Spitzbergen^11^; and the Gartnerkoefel GK-1 core from the Carnic Alps^12^ (Fig. 1b–e). We propose a relationship between the nickel anomaly and the timing of the Siberian Traps eruptions.

In Spitzbergen, (Fig. 1b) nickel spikes of ~100 ppm over a background of about 50 ppm occur at the latest Permian extinction level^11^. Nickel levels in Spitzbergen are generally greater in the early Triassic than in the latest Permian. In the Idrijka Valley, Slovenian section, the latest Permian extinction level is represented by a clayey marl layer with a maximum thickness of 0.8 cm. Nickel is somewhat enriched in the layer to 12 ppm, over a background averaging about 3 ppm^10^ (Fig. 1c). A core section in southern Israel shows greatly enhanced nickel (800 ppm over a background of only about 45 ppm) just below the palynological turnover and fungal event and a negative shift in δ^13^C that mark the P-T boundary^9^ (Fig. 1d). In the Gartnerkoefel GK-1 core, there are two nickel peaks, 110 ppm and 107 ppm, that occur at the times of two negative excursions of δ^13^C that bracket the P-T boundary^12^. Background Ni concentrations in that section of the core are as low as 7 ppm (Fig. 1e) (see Supplementary Information).

In these sections, as well as others, peak nickel values may have been missed as a result of relatively wide sampling of narrow peaks. Absolute values of the peaks vary, possibly because of inhomogeneous areal distribution of nickel-rich volatiles^4^, differences in sedimentation rates and bioturbation^13^, and oxic vs. anoxic depositional conditions^14^. For example, in several of the sections Ni may be secondarily concentrated at redox boundaries, as other redox-sensitive elements (e.g., V, Cr, Co) are also elevated at the nickel peaks^10–12^. Under anoxic or dysoxic conditions (such as indicated by pyrite deposition), nickel will be quickly scavenged from seawater and enriched in the sediments^12^. Other probable volcanogenic elements (e.g., As, Cu, Pb, Sb, Zn) also show peaks correlating with the Ni peak^10–12^. Recent study of Zn concentrations and zinc-isotopic data from the Meishan locality gives further evidence for massive volcanism at the time of the end-Permian extinction. The rapid shift in zinc isotopes suggest that zinc (along with other metals such as nickel) might be coming from volcanic ashes, hydrothermal input and/or extremely fast weathering of Siberian basalts^15^.

## Results

**Figure 2:**
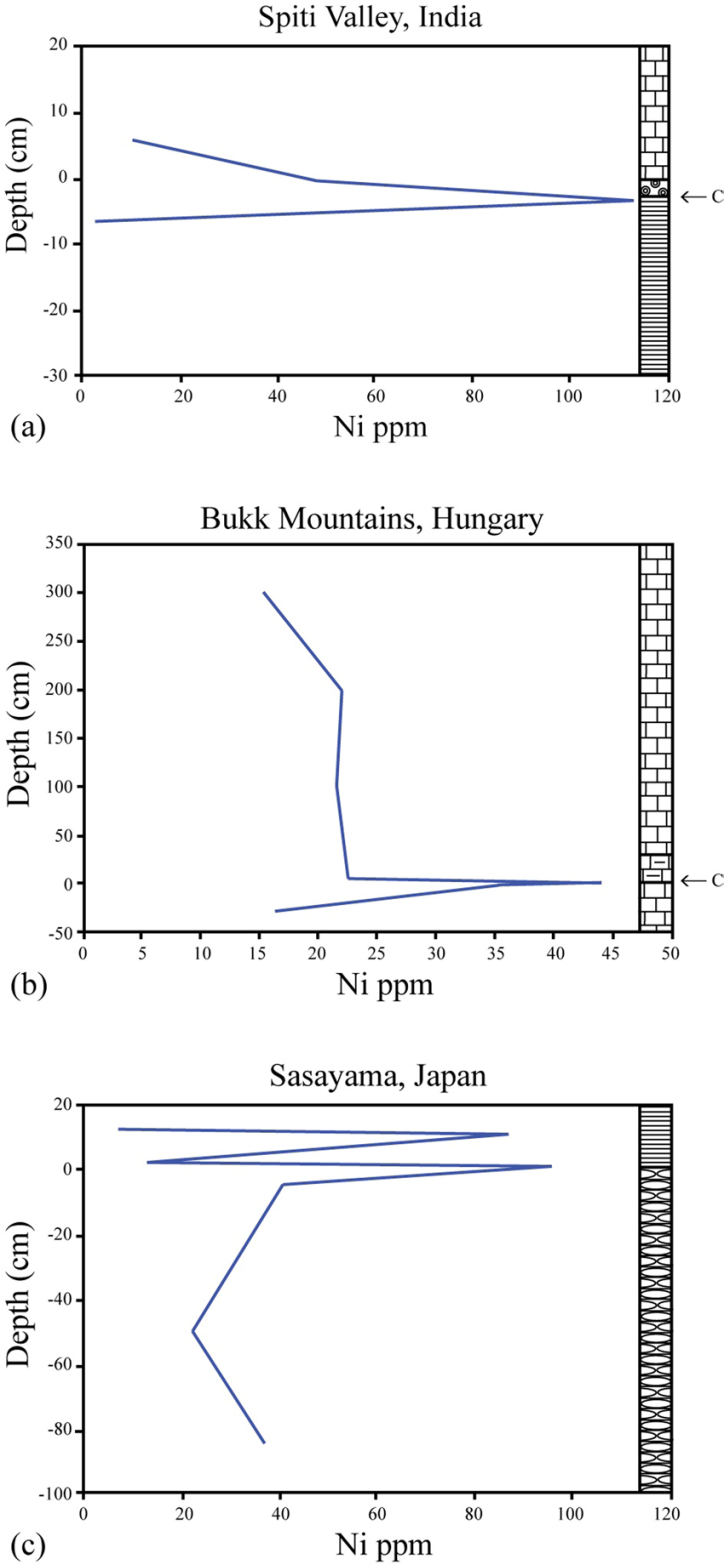
Nickel concentrations at the end of the Permian as determined by ICP-MS analyses by the authors. C marks the position of the end-Permian negative shift in δ^13^C. (**a**) Spiti, India (zero marks the latest Permian extinction layer just below the iron-rich layer and the negative shift in δ^13^C) (4 pts.); (**b**) Bukk Mountains, Hungary (zero marks the latest Permian extinction layer and the negative shift in δ^13^C) (9 pts.); and (**c**) Sasayama, Japan (zero marks the latest Permian extinction level, and the transition from radiolarian chert to black mudstone) (7 pts.). No carbon-isotope data are available for this section (see text for details).

Using solution ICP-MS, with standard addition method (see Methods section), we analyzed samples from P-T boundary sections in Japan (Sasayama), Hungary (Bukk Mountains), and from Spiti, India (Fig. 2). In Japan, the latest Permian extinction level (as determined by radiolarians and other taxa) is marked by a sudden shift from radiolarian cherts to organic-rich black mudstone^16–18^. This sharp transition is close to the P-T boundary as defined by conodonts and radiolarians^16^. At Sasayama, there are two closely spaced nickel peaks of about 86 and 96 ppm over a background of about 20 ppm, within 10 cm of the chert/black shale contact (Fig. 2c). The Hungary section^19,20^ has a thin reddish clay layer at the level of the extinctions and δ^13^C anomaly, with elevated nickel concentrations of 45 ppm over a background of about 20 ppm (Fig. 2b). In India, the boundary interval is marked by a 1 to 5-cm thick ferruginous layer^21,22^, and a negative excursion in δ^13^C (ref.^23^). We found a nickel anomaly of 85 ppm just below the iron-rich layer, over a background of 25 to 50 ppm (Fig. 2a). This suggests a worldwide distribution of the nickel, as India was in the Southern Hemisphere at the time. In these sections, Cr, V and Co also show elevated concentrations at the Ni peak, suggesting deposition under anoxic conditions.

### Nickel anomalies and the Siberian Traps

The Ni increases are interpreted here as reflecting input from volcanic activity of the Siberian Traps^24^, with volatile plumes that may have exhibited a Ni-rich signature^25,26^. The nickel-rich intrusions associated with the Siberian Traps eruptions contain some of the largest deposits of that metal in the world. The release of nickel-rich volatiles is supported by the fact that some Siberian basaltic flows are depleted in Ni and PGEs^25^. This may be a function of segregation of co-existing sulfide liquids in the magma chamber. The sulfides become enriched in the metals, while the silicate magma left behind is depleted in PGEs and Ni. Sulfur volatiles, along with the trace metals would be released in the explosive volcanic plumes^26,27^. Models suggest that explosive gas emissions from the Siberian Traps^26^, and from reactions between related nickel-rich intrusions and coal deposits^5^, could have had a global, though irregular distribution^4^. Widespread dissemination of the volcanic emissions is also supported by the presence of mercury^28^, and fly ash^29^, inferred to be from coal combustion in Siberia, in Permian-Triassic boundary sections far from the eruption site.

### Platinum group elements

Platinum group elements are also somewhat elevated near the P-T boundary. The nickel abundance anomaly at Spiti is associated with a small iridium anomaly (73 ppt) in the ferruginous layer marking the P-T boundary, and another small iridium anomaly (110 ppt) has been detected 70 cm below that layer^21^. A similar double iridium anomaly bracketing the Permian-Triassic boundary is found in the GK-1 core^30^. In that core, the iridium peaks of 165 and 230 ppt, coincide with the two Ni-rich layers, and with two negative δ^13^C anomalies, separated by an estimated 300 to 400 kyr (ref.^31^) (Fig. 1). In a study of osmium-isotopes in the GK-1 core, Koeberl *et al*.^32^ confirmed that these PGE anomalies are most likely terrestrial, not cosmic, in origin. The elevated platinum group metals at the P-T boundary have been attributed to emissions from the Siberian Traps^33^ and such relatively small iridium anomalies could be created by volcanic plumes somewhat enriched in iridium^34,35^.

### Nickel anomalies and isotope shifts

The Ni anomalies are correlated with negative shifts in carbon isotopes in carbonates and organic carbon. These negative δ^13^C anomalies may partly reflect massive releases of light ^12^C (possibly as methane) into the atmosphere, from reactions of magmatic intrusions with carbon-rich sediments^26,36^. It has also been suggested that additional methane came from the successful evolution and rapid expansion of certain methanogenic archaea (e.g., *Methanosarcina)* in the latest Permian seas, driven in part by the increased availability of dissolved nickel in the oceans (which is necessary for methanogenic activities)^7,37^. Negative shifts in δ^18^O at the same time may indicate methane-greenhouse driven increases in global surface temperatures (increases estimated at 8°C) (refs^38,39^). The nickel anomalies may also provide a timeline for correlating the various Permian-Triassic boundary sections.

Furthermore, residence times of volcanogenic methane in the atmosphere and oceans could have been enhanced in the latest Permian. In the modern ocean, the major sinks for methane are reaction with dissolved oxygen, and anaerobic oxidation by sulfate. In the latest Permian, it seems that large portions of the oceans were anoxic or dysoxic (reflected in widespread deposition of organic-rich sediments)^14,40,41^, and atmospheric oxygen is reported to have been less than 16% (ref.^42^). Oceanic sulfate may have been reduced as a result of the widespread formation of pyrite in the anoxic marine environments^43^. Thus, the decrease of the two major oxidants in seawater (oxygen and sulfate), would have decreased aerobic and anaerobic CH_4_ oxidation, and allowed for the enhanced accumulation of CH_4_ in the oceans and atmosphere, contributing to the observed severe global warming at the time of the latest Permian extinction^38,39^.

## Methods

Sample preparation and analyses were performed at Lamont-Doherty Earth Observatory. Samples were powdered using a ball mill with alumina vessels. Precisely weighed sample powders were dissolved at 175 °C in Savillex Teflon beakers on the hotplate in a PicoTrace ® ultra clean laboratory with a mixture of ultrapure nitric, hydrofluoric and perchloric acids. After overnight heating, additional perchloric acid was added if any dark precipitates were observed, which could indicate un-dissolved organic matter. After all sample powders have fully reacted with the acids, the solution was evaporated to dryness at 200 °C in a laminar flow dry-down box. The samples were re-dissolved in nitric acid and evaporated again to convert insoluble fluorides into soluble nitrates. Millipore water was used to rinse perchloric acid condensates down from the inside walls of the beakers, and the resulting slurry was evaporated until the samples dried to flat cakes, and no perchloric acid condensates were observed in the beakers. Finally, 4 N nitric acid was added to the samples to generate a clear solution with no observable precipitates.

Following dissolution, the samples, procedural blanks and international standards, were diluted 2,000 times using 3% nitric acid spiked with 1ppb Indium (In), and analyzed for elemental concentrations using a VG-ExCell® quadrupole ICP-MS. In-run oxide formation was monitored using CeO/Ce, and maintained below 3%. Interference of BaO ions on Eu is corrected using the observed correlation between ^153^Eu and ^137^Ba measured on pure Ba solutions, during the same run session. Drift corrections were made based on repeated measurements of a drift solution mixed from representative sample solutions. After external drift correction, a secondary internal drift correction was applied using In. Final elemental concentrations are determined using the standard addition method. One SD relative uncertainties for all elements are better than 2%. While procedural blanks are generally below 2%, the minimum counts for 6 procedural blank solutions were subtracted from the raw counts of the unknowns.

## Electronic supplementary material

Dataset 1
