# Supplementary material for: Global nickel anomaly links Siberian Traps eruptions and the latest Permian mass extinction
Source: Sci Rep. 2017 Sep 29;7:12416. doi: 10.1038/s41598-017-12759-9 (PMC5622041; doi:10.1038/s41598-017-12759-9)
Supplement: Supplementary file 1 — Dataset 1 [file 41598_2017_12759_MOESM1_ESM.doc]

**Global nickel anomaly links Siberian Traps eruptions and the latest Permian mass extinction**

Michael R. Rampino^1,2,3*^ , Sedelia Rodriguez^4^, Eva Baransky^4^ & Yue Cai^5^

^1^Department of Biology, New York University, New York, NY 10003, USA

^2^Department of Environmental Studies, New York University, New York, NY 10003, USA

^3^NASA, Goddard Institute for Space Studies, New York, NY 10025, USA

^4^Department of Environmental Science, Barnard College, New York, NY 10027, USA

^5^Lamont-Doherty Earth Observatory of Columbia University, Palisades, NY 10964, USA

*Correspondence to Rampino: [mrr1@nyu.edu](mailto:mrr1@nyu.edu)

| **HUNGAR** | **Y** |  |  |  |  |  |  |  |  |
| --- | --- | --- | --- | --- | --- | --- | --- | --- | --- |
| **Sample** | **GER1** | **GER2** | **GER3** | **GER4** | **GER5** | **GER6** | **GER7** | **GER8** | **GER9** |
| **Depth (m)** | -0.3 | 0 | 0.01 | 0.02 | 0.03 | 0.05 | 1 | 2 | 3 |
| **Li** | 1.5 | 39.8 | 49.9 | 30.3 | 17.5 | 14.2 | 20.6 | 16.7 | 1.7 |
| **Be** | 0.2 | 1.2 | 1.7 | 0.9 | 0.6 | 0.5 | 0.7 | 0.7 | 0.1 |
| **Sc** | 0.6 | 7.7 | 8.7 | 4.3 | 3.1 | 2.9 | 3.7 | 4.5 | 0.7 |
| **V** | 17.7 | 92.7 | 105.3 | 63.3 | 51.0 | 47.8 | 50.4 | 44.2 | 12.6 |
| **Cr** | 4.8 | 87.7 | 97.7 | 49.4 | 34.2 | 29.3 | 22.7 | 27.0 | 3.4 |
| **Co** | 2.3 | 4.9 | 7.6 | 4.5 | 4.9 | 3.7 | 4.5 | 4.6 | 2.1 |
| **Ni** | 15.4 | 34.8 | 41.4 | 22.0 | 22.6 | 21.5 | 20.3 | 20.5 | 13.8 |
| **Cu** | 3.5 | 8.7 | 10.3 | 5.3 | 7.3 | 7.0 | 7.6 | 6.3 | 3.5 |
| **Zn** | 34.4 | 37.4 | 50.3 | 24.5 | 27.0 | 26.4 | 34.9 | 18.6 | 12.8 |
| **Rb** | 3.6 | 126.8 | 139.0 | 121.2 | 39.2 | 29.9 | 39.8 | 56.7 | 3.1 |
| **Sr** | 1854 | 1059 | 905 | 453 | 2392 | 2847 | 2012 | 972 | 1184 |
| **Y** | 1.2 | 7.6 | 9.0 | 4.2 | 9.8 | 8.9 | 28.3 | 27.8 | 3.2 |
| **Nb** | 0.3 | 5.1 | 6.1 | 3.7 | 2.3 | 1.8 | 2.8 | 3.9 | 0.6 |
| **Mo** | 0.3 | 0.6 | 1.0 | 0.7 | 0.7 | 0.6 | 0.4 | 0.2 | 0.1 |
| **Cd** | 0.0 | 0.0 | 0.0 | 0.0 | 0.0 | 0.0 | 0.0 | 0.0 | 0.0 |
| **Cs** | 0.2 | 5.1 | 9.1 | 8.3 | 1.6 | 1.0 | 1.5 | 2.2 | 0.1 |
| **Ba** | 8.8 | 218.5 | 237.0 | 116.3 | 56.9 | 45.3 | 61.8 | 83.7 | 8.8 |
| **La** | 1.1 | 7.5 | 11.4 | 4.5 | 12.5 | 7.6 | 23.5 | 34.2 | 2.0 |

| **HUNGARY** | |  | |  | |  | |  | |  | |  | |  | |  | |  | |
| --- | --- | --- | --- | --- | --- | --- | --- | --- | --- | --- | --- | --- | --- | --- | --- | --- | --- | --- | --- |
| **Sample** | **GER1** | | **GER2** | | **GER3** | | **GER4** | | **GER5** | | **GER6** | | **GER7** | | **GER8** | | **GER9** | |  |
| **Depth (m)** | -0.3 | | 0 | | 0.01 | | 0.02 | | 0.03 | | 0.05 | | 1 | | 2 | | 3 | |  |
| **Ce** | 1.5 | | 17.3 | | 22.0 | | 9.0 | | 21.7 | | 16.9 | | 31.7 | | 40.4 | | 3.1 | |  |
| **Pr** | 0.3 | | 2.0 | | 2.6 | | 1.2 | | 2.4 | | 1.8 | | 4.4 | | 5.9 | | 0.5 | |  |
| **Nd** | 0.7 | | 7.0 | | 9.0 | | 4.2 | | 8.1 | | 6.1 | | 16.7 | | 21.8 | | 1.7 | |  |
| **Sm** | 0.2 | | 1.4 | | 1.8 | | 0.8 | | 1.6 | | 1.2 | | 3.4 | | 4.1 | | 0.4 | |  |
| **Eu** | 0.0 | | 0.3 | | 0.3 | | 0.1 | | 0.4 | | 0.4 | | 1.1 | | 0.8 | | 0.1 | |  |
| **Tb** | 0.0 | | 0.2 | | 0.2 | | 0.1 | | 0.2 | | 0.2 | | 0.5 | | 0.6 | | 0.1 | |  |
| **Gd** | 0.1 | | 1.3 | | 1.6 | | 0.7 | | 1.5 | | 1.2 | | 3.5 | | 3.9 | | 0.4 | |  |
| **Dy** | 0.1 | | 1.3 | | 1.6 | | 0.7 | | 1.3 | | 1.1 | | 3.0 | | 3.3 | | 0.4 | |  |
| **Ho** | 0.0 | | 0.3 | | 0.3 | | 0.1 | | 0.3 | | 0.2 | | 0.6 | | 0.6 | | 0.1 | |  |
| **Er** | 0.1 | | 0.8 | | 0.9 | | 0.4 | | 0.7 | | 0.6 | | 1.6 | | 1.7 | | 0.2 | |  |
| **Tm** | 0.0 | | 0.1 | | 0.1 | | 0.1 | | 0.1 | | 0.1 | | 0.2 | | 0.2 | | 0.0 | |  |
| **Yb** | 0.1 | | 0.8 | | 1.0 | | 0.5 | | 0.6 | | 0.5 | | 1.2 | | 1.4 | | 0.2 | |  |
| **Lu** | 0.0 | | 0.1 | | 0.1 | | 0.1 | | 0.1 | | 0.1 | | 0.2 | | 0.2 | | 0.0 | |  |
| **Ta** | 0.0 | | 0.4 | | 0.5 | | 0.1 | | 0.2 | | 0.1 | | 0.2 | | 0.3 | | 0.0 | |  |
| **Tl** | 0.0 | | 0.5 | | 0.7 | | 0.6 | | 0.2 | | 0.1 | | 0.2 | | 0.2 | | 0.0 | |  |
| **Pb** | 2.3 | | 13.0 | | 16.8 | | 9.9 | | 8.4 | | 101.8 | | 6.4 | | 3.6 | | 3.2 | |  |
| **Th** | 0.2 | | 4.3 | | 5.3 | | 2.5 | | 1.6 | | 1.4 | | 2.2 | | 3.5 | | 0.3 | |  |
| **U** | 2.6 | | 2.8 | | 3.3 | | 1.6 | | 4.0 | | 5.2 | | 4.2 | | 1.7 | | 0.4 | |  |

| **INDIA** |  |  |  |  |
| --- | --- | --- | --- | --- |
| **Sample** | **LL4** | **LL10B** | **LL10C** | **LL11** |
| **Depth (m)** | 0.06 | 0 | -0.03 | -0.06 |
| **Li** | 9.8 | 14.7 | 18.3 | 54.6 |
| **Be** | 3.3 | 3.6 | 4.4 | 2.2 |
| **Sc** | 19.4 | 16.3 | 12.4 | 7.0 |
| **V** | 170.1 | 182.0 | 145.6 | 68.4 |
| **Cr** | 110.8 | 124.6 | 124.9 | 30.4 |
| **Co** | 21.3 | 8.4 | 50.7 | 41.6 |
| **Ni** | 36.8 | 25.8 | 85.2 | 54.7 |
| **Cu** | 22.2 | 25.1 | 27.4 | 77.7 |
| **Zn** | 115.3 | 118.1 | 169.5 | 163.7 |
| **Rb** | 217.0 | 208.1 | 202.9 | 59.5 |
| **Sr** | 149 | 65 | 77 | 2506 |
| **Y** | 23.0 | 15.6 | 15.9 | 305.0 |
| **Nb** | 12.9 | 25.7 | 7.5 | 3.1 |
| **Mo** | 0.7 | 2.8 | 1.2 | 12.3 |
| **Cd** | 0.1 | 0.1 | 0.1 | 0.1 |
| **Cs** | 19.4 | 13.8 | 13.7 | 2.6 |
| **Ba** | 800.0 | 776.7 | 754.8 | 257.7 |
| **La** | 52.3 | 38.0 | 31.7 | 156.9 |

| **INDIA** |  |  |  |  |
| --- | --- | --- | --- | --- |
| **Sample** | **LL4** | **LL10B** | **LL10C** | **LL11** |
| **Depth (m)** | 0.06 | 0 | -0.03 | -0.06 |
| **Ce** | 121.5 | 74.9 | 54.0 | 440.1 |
| **Pr** | 12.0 | 5.7 | 4.0 | 50.4 |
| **Nd** | 39.2 | 19.2 | 13.3 | 244.1 |
| **Sm** | 6.7 | 2.9 | 2.1 | 62.1 |
| **Eu** | 1.1 | 0.7 | 0.6 | 27.5 |
| **Tb** | 0.7 | 0.4 | 0.4 | 8.8 |
| **Gd** | 5.0 | 2.6 | 2.3 | 63.7 |
| **Dy** | 4.3 | 2.6 | 2.4 | 50.3 |
| **Ho** | 0.8 | 0.6 | 0.5 | 9.1 |
| **Er** | 2.4 | 1.7 | 1.7 | 21.7 |
| **Tm** | 0.4 | 0.3 | 0.3 | 2.3 |
| **Yb** | 2.5 | 2.0 | 2.0 | 12.7 |
| **Lu** | 0.4 | 0.3 | 0.3 | 1.6 |
| **Ta** | 0.7 | 1.5 | 0.5 | 0.2 |
| **Tl** | 1.0 | 1.0 | 1.1 | 0.3 |
| **Pb** | 18.2 | 28.2 | 51.4 | 77.9 |
| **Th** | 23.6 | 24.6 | 24.1 | 8.6 |
| **U** | 2.7 | 4.2 | 4.4 | 28.1 |

| **SASAYAMA** |  |  |  |  |  |  |  |
| --- | --- | --- | --- | --- | --- | --- | --- |
| **Sample** | **SAS1** | **SAS2** | **SAS3** | **SAS4** | **SAS5** | **SAS6** | **SAS7** |
| **Depth (m)** | 0.125 | 0.1 | 0.02 | 0 | -0.05 | -0.5 | -0.85 |
| **Li** | 9.8 | 27.3 | 11.5 | 27.0 | 11.4 | 7.0 | 10.0 |
| **Be** | 0.2 | 2.2 | 0.9 | 2.6 | 0.6 | 0.4 | 0.7 |
| **Sc** | 1.0 | 8.7 | 3.5 | 11.2 | 5.1 | 2.2 | 6.3 |
| **V** | 8.8 | 371.1 | 46.0 | 353.1 | 124.0 | 65.5 | 126.3 |
| **Cr** | 5.4 | 107.5 | 18.9 | 127.4 | 24.4 | 7.7 | 40.2 |
| **Co** | 0.6 | 10.9 | 12.9 | 15.7 | 12.9 | 8.0 | 8.7 |
| **Ni** | 5.8 | 84.1 | 11.3 | 93.5 | 39.5 | 20.8 | 35.2 |
| **Cu** | 13.2 | 46.7 | 49.7 | 33.9 | 23.0 | 20.0 | 23.5 |
| **Zn** | 7.6 | 193.9 | 31.1 | 187.7 | 38.1 | 21.2 | 55.2 |
| **Rb** | 5.7 | 83.0 | 37.1 | 104.7 | 25.5 | 11.6 | 31.1 |
| **Sr** | 5 | 22 | 17 | 27 | 10 | 10 | 17 |
| **Y** | 5.5 | 22.9 | 10.2 | 36.0 | 12.3 | 3.0 | 17.1 |
| **Nb** | 0.9 | 9.6 | 3.3 | 14.5 | 5.5 | 1.5 | 8.4 |
| **Mo** | 1.0 | 0.6 | 11.6 | 0.3 | 0.6 | 0.4 | 2.2 |
| **Cd** | 0.0 | 0.1 | 0.0 | 0.2 | 0.0 | 0.0 | 0.1 |
| **Cs** | 0.3 | 3.8 | 1.4 | 5.3 | 1.2 | 0.7 | 1.7 |
| **Ba** | 19.8 | 200.7 | 133.8 | 236.5 | 76.0 | 52.8 | 94.3 |
| **La** | 3.7 | 31.3 | 14.4 | 39.7 | 15.0 | 4.2 | 19.3 |

| **SASAYAMA** |  |  |  |  |  |  |  |
| --- | --- | --- | --- | --- | --- | --- | --- |
| **Sample** | **SAS1** | **SAS2** | **SAS3** | **SAS4** | **SAS5** | **SAS6** | **SAS7** |
| **Depth (m)** | 0.125 | 0.1 | 0.02 | 0 | -0.05 | -0.5 | -0.85 |
| **Ce** | 11.0 | 58.0 | 25.4 | 79.6 | 42.6 | 14.8 | 56.6 |
| **Pr** | 0.9 | 6.6 | 2.5 | 9.0 | 2.9 | 0.9 | 3.9 |
| **Nd** | 3.9 | 22.7 | 9.0 | 33.1 | 11.1 | 3.5 | 14.9 |
| **Sm** | 0.8 | 3.9 | 1.7 | 6.4 | 1.9 | 0.6 | 2.5 |
| **Eu** | 0.2 | 0.8 | 0.4 | 1.3 | 0.3 | 0.1 | 0.5 |
| **Tb** | 0.1 | 0.5 | 0.2 | 0.9 | 0.3 | 0.1 | 0.3 |
| **Gd** | 1.0 | 3.4 | 1.6 | 6.0 | 1.6 | 0.5 | 2.2 |
| **Dy** | 0.7 | 3.3 | 1.5 | 5.2 | 1.7 | 0.5 | 2.3 |
| **Ho** | 0.1 | 0.7 | 0.3 | 1.1 | 0.4 | 0.1 | 0.6 |
| **Er** | 0.4 | 2.2 | 0.8 | 3.0 | 1.2 | 0.3 | 1.8 |
| **Tm** | 0.0 | 0.3 | 0.1 | 0.4 | 0.2 | 0.0 | 0.3 |
| **Yb** | 0.3 | 2.2 | 0.8 | 2.9 | 1.2 | 0.3 | 2.0 |
| **Lu** | 0.0 | 0.3 | 0.1 | 0.4 | 0.2 | 0.0 | 0.3 |
| **Ta** | 0.0 | 0.1 | 0.2 | 0.9 | 0.1 | 0.1 | 0.2 |
| **Tl** | 0.1 | 0.9 | 0.4 | 1.1 | 0.3 | 0.1 | 0.5 |
| **Pb** | 1.6 | 18.2 | 6.8 | 23.4 | 4.1 | 3.5 | 4.8 |
| **Th** | 0.6 | 8.8 | 2.8 | 10.5 | 3.6 | 1.0 | 6.2 |
| **U** | 0.2 | 4.7 | 1.5 | 5.8 | 2.3 | 0.8 | 4.2 |
